# Multi-objective representation learning for road networks and trajectories with spatial-temporal fusion and contrastive signals

**DOI:** 10.1371/journal.pone.0331473

**Published:** 2025-09-18

**Authors:** Ashraful Islam Shanto Sikder, Naushin Nower

**Affiliations:** Institute of Information Technology, University of Dhaka, Dhaka, Bangladesh; National University of Defense Technology, CHINA

## Abstract

Modeling and learning representations for road networks and vehicle trajectories are crucial in enabling intelligent transportation systems, with applications ranging from traffic forecasting to many other downstream inference tasks. However, learning effective representations that generalize well across tasks remains challenging due to the heterogeneous nature of spatio-temporal data and limited supervision. In this paper, we propose a unified multi-objective pretraining framework called MRRT, **M**ulti-objective **R**epresentation Learning for **R**oad Network and **T**rajectories, that combines masked trajectory modeling (MTM) with multiple contrastive learning objectives across trajectories, road segments, and spatial contexts. Our model integrates graph attention networks (GAT), spatial CNNs, and transformers with temporal and positional encoding, allowing us to capture structural and contextual dependencies in urban mobility. By leveraging grid-structured and graph-structured data, along with spatiotemporal dynamics, our model effectively captures diverse road and trajectory characteristics. To enhance robustness, we design trajectory-specific data augmentations and contrastive heads for trajectory-to-trajectory, trajectory-to-node, and node-to-node alignment. Additionally, we design an adaptive negative sampling strategy to further enhance the contrastive learning. We evaluate our approach in various downstream tasks based on trajectory and road, including travel time estimation, speed inference, and similarity search. Extensive experiments demonstrate that our method consistently outperforms prior baselines and ablated variants, validating the effectiveness of our multiobjective design.

## Introduction

With the rapid proliferation of GPS-enabled devices and the increasing availability of large-scale mobility data, representation learning for road networks and vehicle trajectories has become a cornerstone of intelligent transportation systems (ITS). These representations serve as compact, expressive encodings of complex spatio-temporal patterns and are widely adopted in a range of downstream tasks, such as travel time estimation [1], traffic speed inference [2], route recommendation [3], and trajectory similarity retrieval [2]. Effective representation learning can reduce the dependence on task-specific feature engineering and enable generalization across diverse cities and applications [4,5].

A road network is typically modeled as a graph, where nodes represent road segments and edges encode their topological relationships. Conversely, a trajectory represents a temporally ordered sequence of traversed segments, capturing dynamic mobility semantics across both space and time. While the road network provides a static structural view of the urban infrastructure, trajectories reflect time-varying behavioral patterns influenced by user preferences, traffic dynamics, and regional contexts. Capturing the interaction between these two complementary modalities is essential for learning holistic spatiotemporal representations.

Early approaches to representation learning in this domain focused on a single modality. For instance, Node2vec [6] employs biased random walks and skip-gram models to learn node embeddings, ignoring the real-world sequential movements observed through trajectories. T2vec [7] focuses on learning trajectory representations through a spatial-proximity aware encoder-decoder mechanism, but does not consider the underlying topological structure as well as the node features of the road network. These models fall short in modeling inter-modal dependencies, leading to incomplete representations. More recent efforts [8] adopt two-stage pipelines, where representations from one modality (e.g., road network) are first learned and then injected into the other (e.g., trajectory) through sequential transfer. Although this improves semantic enrichment, it introduces three key limitations: (1) *Error propagation* between stages can undermine representation quality, (2) *Insufficient cross-scale interaction modeling* fails to unify local (road) and global (trajectory) semantics effectively, and (3) *Temporal dynamics*—including periodicity, burstiness, and congestion effects - are oversimplified or ignored.

In contrast, end-to-end joint learning frameworks like JCLRNT [2] demonstrate that simultaneous optimization of both road and trajectory representations via contrastive objectives leads to improved downstream performance. These methods exploit the mutual dependencies between road-level and trajectory-level data to enhance representation fidelity. In addition to employing contrastive objectives, TCRTRL [9] incorporates a hard negative sampling module for generating harder synthetic negatives and thus enhancing the discriminative capability. However, these methods still rely solely on discriminative contrastive objectives and do not incorporate generative signals such as masked modeling, which can enhance contextual understanding. RED [4] integrates masked trajectory modeling and next-segment prediction but remains restricted to single-modality (trajectory-only) learning and lacks graph-level spatial awareness. Moreover, other recent methods like GREEN [10] leverage dual-modal trajectory encoding (road vs. grid), but the fusion occurs post-encoding, limiting fine-grained interaction. Temporal modeling is often limited to static or hand-engineered encoding, as in DyToast [5], which uses fixed trigonometric functions to represent time.

To address these limitations, we propose a unified, multi-modal representation learning framework that learns robust, general-purpose embeddings for both road networks and trajectories using Masked Trajectory Modeling and Contrastive Learning. Masked modeling, being a generative style objective, requires the encoder to reason over contextual dependencies, while contrastive learning, being a discriminative objective, enforces semantic consistency. Combining both in a unified training pipeline allows our model to learn richly contextualized and transferable representations. This joint optimization not only mitigates error propagation but also enables the model to leverage both local structure and global dynamics effectively. This joint formulation has proven effective in other domains such as NLP and vision, where models like BERT [11] and SimCLR [12] demonstrate that generative style pre-training enhances understanding of the fine-grained context, while contrastive objectives improve representation discrimination and robustness. Together, they act as complementary forces, one promoting semantic abstraction, the other improving relational alignment, leading to more versatile and generalizable embeddings.

Our contributions are as follows.

- We design a hybrid architecture that jointly encodes road network topology using Graph Attention Networks (GANs) [13] and dynamic trajectories using transformer-based [14] sequence encoders, enabling multiscale spatiotemporal representation learning.
- We introduce a spatial fusion module that injects grid-based convolutional features into both node- and trajectory-level embeddings via attention-based interaction, enhancing spatial context awareness beyond pure topological structure.
- We adopt a multi-objective training strategy that combines masked trajectory modeling with contrastive learning across road-road, trajectory-trajectory, and road-trajectory pairs, incorporating an adaptive negative sampling strategy, thereby learning both semantic structure and inter-modal alignment.

Extensive experiments on multiple real-world datasets validate the superiority of our model in four downstream tasks, including road classification, traffic speed regression, trajectory similarity search, and travel-time estimation. The results demonstrate that our model significantly outperforms state-of-the-art methods, affirming the effectiveness of joint modeling, spatial fusion, and multi-objective learning.

## Related works

Representation learning in road networks and trajectories has attracted substantial attention in recent years due to its importance in various traffic-related applications. Existing research can be broadly categorized into road network representation learning, trajectory representation learning, and joint approaches that integrate both types of data.

### Road network representation learning

The study of road network representation learning typically aims to capture road segments’ structural and functional properties. Traditional graph embedding methods, such as Node2Vec [6], employ biased random walks and skip-gram models to learn node embeddings, making them general-purpose but often insufficient for road-specific tasks. Similarly, DGI (Deep Graph Infomax) [15] uses unsupervised learning to maximize mutual information between local and global graph representations, but lacks explicit road and traffic-specific adaptations. More specialized methods have been developed to address these limitations. For example, RFN (Relational Fusion Networks) [3] introduces a more targeted approach, modeling interactions among nodes and edges through relational views and message passing. IRN2Vec [16] focuses on capturing relationships between pairs of road segments using samples from the shortest routes, improving the embedding process by incorporating task-related information through multi-objective learning. HRNR (Hierarchical Road Network Representation) [17] advances these efforts by employing a hierarchical GNN [18] architecture to embed functional and structural properties at multiple levels: from road segments to larger structural regions. Despite these advancements, many of these methods either neglect trajectory data or only utilize it in isolated post-processing steps, missing out on potentially mutually beneficial learning between road segments and traffic movement.

### Trajectory representation learning

Trajectory representation learning methods primarily focus on modeling sequential movement data for downstream tasks such as travel time prediction and similar trajectory search. T2Vec [7] takes an approach employing an encoder-decoder structure with LSTM [19] units to handle noisy trajectory sequences and reconstruct trajectories to enhance representation learning. Advanced methods such as Toast [20] go further by integrating road network context with trajectory data, applying a Transformer-based module to incorporate auxiliary traffic information. This multi-step approach has demonstrated success in improving trajectory-based task performance. Similarly, GTS (Graph Trajectory Similarity) [21] combines POI embeddings and GNN-LSTM networks to represent trajectories by learning both point-wise and sequence-level dependencies. Although these approaches address trajectory representation to varying degrees, they often do so without a unified approach that fully integrates road network data, which can lead to suboptimal performance in downstream applications.

### Road and trajectory representation learning

There have recently been efforts to create integrated models that leverage road and trajectory data to embed interconnected elements simultaneously. Joint Contrastive Learning of Road Network and Trajectories (JCLRNT) [2] explores joint contrastive learning of road networks and trajectories by aligning their embedding spaces. However, it primarily focuses on contrastive alignment without effectively modeling the spatio-temporal dependencies within the trajectories. START [8] also proposes a framework for utilizing the road network and trajectories simultaneously, including temporal embeddings with a minutes index and a day-of-week index. However, it focuses only on trajectory representation learning. LightPath [22] presents a lightweight, path-based representation learning approach to reduce the computational cost of trajectory modeling. Although it achieves state-of-the-art efficiency, its performance is heavily dependent on heuristic-based feature extraction, which limits its ability to learn complex mobility patterns in data-driven environments.

The above discussed state-of-the-art studies highlight the importance of integrating road network and trajectory learning, but they exhibit several limitations: (1) Insufficient modeling of fine-grained spatio-temporal interactions between road segments and trajectories; (2) Most models either use contrastive learning or masked modeling in isolation, rather than integrating both to leverage complementary strengths; and (3) Reliance on static or random negative sampling strategies for contrastive learning, which can hinder learning by including semantically trivial negatives.

Our proposed model addresses these limitations through a unified transformer-based framework that performs multi-level fusion of road and trajectory information using positional encodings, temporal embeddings, and cross-attention-based spatial fusion. Beyond conventional contrastive objectives, we introduce an adaptive negative sampling strategy that enhances representation discrimination by dynamically retrieving hard negatives using Approximate Nearest Neighbor (ANN) [23] search. This approach is further strengthened through curriculum-aware difficulty scheduling and negative mixing, inspired by MOCHI [24], which progressively challenges the model throughout training. The combination of structural modeling, temporal awareness, and adaptive contrastive sampling allows our method to achieve a stronger generalization across multiple downstream tasks, including road classification, speed inference, travel time estimation, and trajectory similarity search.

## Notations and definitions

In this section, we introduce the notation and preliminaries, followed by the formal definition of the problem. Scalars are represented in italics (e.g., *n*), vectors in lowercase boldface (e.g., **h**), matrices in uppercase boldface (e.g., **A**), and sets in script capitals (e.g., 𝒢).

**Road network:** A road network is modeled as a directed graph $𝒢=\langle S,{𝐀}_{s}\rangle$, where *S* is the set of vertices representing road segments, with |S| as the number of segments. The adjacency matrix ${𝐀}_{s}\in {\mathbb{R}}^{\left|S|\times |S\right|}$ has entries ${𝐀}_{s}[s_{i},s_{j}]$ that are binary, indicating whether there is a common intersection between the end of segment *s*_*i*_ and the start of segment *s*_*j*_.

**Trajectory:** A trajectory 𝒯 is a time-ordered sequence of pairs of consecutive road segments and timestamps, represented as $𝒯=[\langle s_{i},t_{i}\rangle {]}_{i=1}^{m}$, where $s_{i}\in S$ denotes the *i*-th road segment in the trajectory, and *t*_*i*_ is the visit timestamp for *s*_*i*_. Trajectories capture the movement of an object within the road network 𝒢.

**Representation learning for road networks and trajectories:** Given a road network $𝒢=\langle S,{𝐀}_{s}\rangle$ and a set of historical trajectories 𝒟, the objective is to learn a representation matrix ${𝐇}_{s}\in {\mathbb{R}}^{|S|\times d}$, where the *i*-th row, ${𝐡}_{s_{i}}$, represents the embedding for road segment *s*_*i*_. Additionally, for each trajectory 𝒯∈𝒟, we aim to learn a representation vector ${𝐡}_{𝒯}\in {\mathbb{R}}^{d}$.

The frequently used symbols in the article are listed in Table 1.

**Table 1:** Frequently used symbols.

Symbol	Description
𝒢	An input road network graph
*S*	The set of road segments in 𝒢
𝒯	A time-ordered sequence of pairs of consecutive road segments and timestamps, $𝒯=[\langle s_{i},t_{i}\rangle ]$
𝒟	The set of all trajectories 𝒯. All 𝒯∈𝒟.
${𝐀}_{s}$	The adjacency matrix of 𝒢
*s*_*i*_	The *i*-th road segment, where $s_{i}\in S$
${𝐇}_{s}$	A representation matrix for the road network 𝒢, with the *i*-th row, ${𝐡}_{s_{i}}$, representing the embedding of the *i*-th road segment in *S*.

## Methodology

This section describes our proposed unified trajectory and road network representation learning framework. The goal is to jointly encode spatial, temporal, and structural information from trajectories and road segments to produce generalizable embeddings for downstream mobility tasks.

### Overall framework

**Fig 1:**
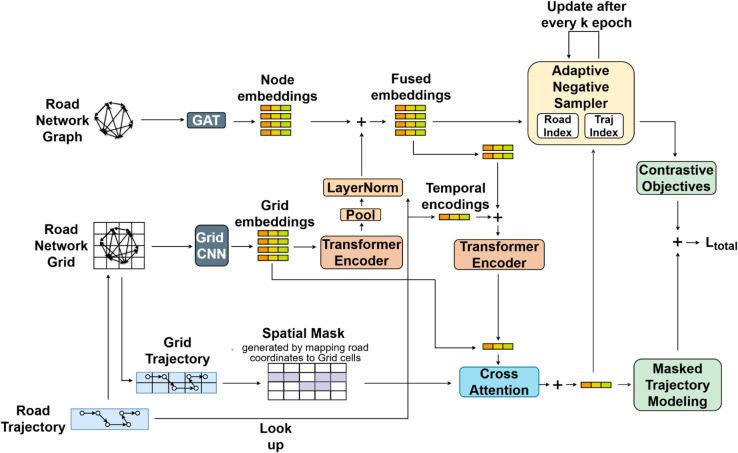
Proposed MRRT model.

Our model takes as input a road network $𝒢=\langle S,{𝐀}_{s}\rangle$ and a trajectory dataset $𝒟=\{{𝒯}_{i}\}$, where each trajectory ${𝒯}_{i}=[\langle s_{1},t_{1}\rangle ,\ldots ,\langle s_{m},t_{m}\rangle ]$ is a sequence of road segments with timestamps. The model learns representations for:

- **Road segments:** capturing both topological structure and contextual semantics;
- **Trajectories:** encoding sequential spatio-temporal patterns across road segments;

The learned embeddings are optimized using a combination of masked modeling and contrastive learning objectives. The high-level model flow is illustrated in Fig 1.

The architecture consists of several modular components - a Graph Attention Network (GAT) encodes road segment connectivity, while a CNN-Spatial Transformer pipeline extracts and models spatial context from a grid-based map partitioning. These representations are fused through residual pooling. Trajectory encoding is performed using a Transformer encoder over temporally enriched node sequences, with a cross-attention mechanism to integrate spatial context. The framework further incorporates two self-supervised losses - Masked Trajectory Modeling and Contrastive Loss- and an adaptive negative sampling strategy that leverages dual FAISS indexes for hard negative selection throughout training.

### Road and spatial representation module

We represent the road network as a graph and use a GAT-based encoder to extract structural representations. Each road segment *s*_*i*_ is either embedded from raw node features ${𝐱}_{i}\in {\mathbb{R}}^{d_{\text{in}}}$ or from a learnable node embedding $\in {\mathbb{R}}^{d}$.

$${𝐇}_{s}=\text{GAT}(𝐗,{𝐀}_{s}),\;{𝐇}_{s}\in {\mathbb{R}}^{|S|\times d}$$ (1)

We introduce a CNN-based [25] grid encoder over the spatial map to capture regional semantics. The spatial grid is constructed by partitioning the city map into a fixed-resolution grid. We extract spatial features using a CNN followed by a spatial transformer encoder:

$${𝐇}_{\text{spatial}}=\text{Transformer}(\text{Flatten}(\text{CNN}(\text{Grid})))$$ (2)

These representations are fused via residual addition with context pooling:

$${𝐇}_{s}^{\prime }={𝐇}_{s}+\text{LayerNorm}(\text{Dropout}(\text{MeanPool}({𝐇}_{\text{spatial}})))$$ (3)

Graph Attention Networks (GATs) are well-suited for modeling relational dependencies between road segments based on network topology, capturing connectivity and local structure. However, GATs alone may struggle to capture broader spatial context—such as regional semantics, urban morphology, or neighborhood-level traffic patterns—which are often reflected in the physical layout of the environment. We introduce the CNN-based encoder over rasterized spatial grids to address this, which excels at learning localized spatial textures and regional patterns due to its inductive bias toward translational invariance. By fusing GAT-derived relational features with CNN-extracted spatial context via residual addition, our model benefits from both structured graph information and dense spatial priors, enabling more context-aware representations. This design is empirically validated in our ablation studies, where the full model (GAT + CNN) consistently outperforms configurations using only GAT, supporting the claim of complementary behavior.

### Trajectory representation module

Given a trajectory $𝒯=[\langle s_{1},t_{1}\rangle ,\ldots ,\langle s_{m},t_{m}\rangle ]$, we obtain the representation ${𝐡}_{s_{i}}$ for each road segment *s*_*i*_ by indexing into the fused graph embeddings:

$${𝐡}_{s_{i}}={𝐇}_{s}^{\prime }[s_{i}]$$ (4)

We enrich each token with temporal signals using temporal encodings and time-aware embeddings:

$${𝐡}_{s_{i}}^{\prime }={𝐡}_{s_{i}}+\text{TemporalEnc}(t_{i})+\text{HourEmb}(t_{i})+\text{WeekdayEmb}(t_{i})$$ (5)

These enriched embeddings are then passed through positional encoding and a transformer encoder to obtain contextualized trajectory representations:

$${𝐇}_{𝒯}=\text{Transformer}(\text{PosEnc}([{𝐡}_{s_{1}}^{\prime },\ldots ,{𝐡}_{s_{m}}^{\prime }]))$$ (6)

To enhance spatial reasoning, we apply cross-attention between trajectory tokens ${𝐇}_{𝒯}$ and the spatial grid ${𝐇}_{\text{spatial}}$ using a trajectory-aware attention mask ${𝐌}_{\text{spatial}}$:

$${𝐇}_{𝒯}^{\prime }={𝐇}_{𝒯}+\text{CrossAttn}({𝐇}_{𝒯},{𝐇}_{\text{spatial}},{𝐇}_{\text{spatial}},{𝐌}_{\text{spatial}})$$ (7)

Here ${𝐌}_{\text{spatial}}\in \{0,1{\}}^{B\times HW}$ is a binary mask that activates only grid cells spatially relevant to the trajectory path. The final trajectory embedding ${𝐡}_{T}$ is pooled via attention-masked average pooling over the encoded tokens in ${𝐇}_{𝒯}^{\prime }$.

### Self-supervised learning objectives

We apply multi-objective self-supervised learning to optimize representations of road segments and trajectories. Four key objectives are used in training:

**(1) Masked Trajectory Modeling (MTM):** A subset of trajectory positions is masked using a learned token. The model is trained to reconstruct the masked road segments using a softmax classification head:

$${ℒ}_{\text{MTM}}=\text{CrossEntropy}(𝐖\cdot {𝐡}_{\text{masked}},{𝐲}_{\text{true}})$$ (8)

Before moving on to the contrastive objectives, we define a unified notation to distinguish between trajectory-level and road-level representations used in our contrastive and reconstruction-based learning framework.

Let ${𝐇}_{s}^{\prime }$ denote the final matrix of road segment embeddings and ${𝐇}_{𝒯}^{\prime }$ the encoded matrix of trajectory token embeddings. We define

- ${𝐞}_{r}$: the embedding of road segment *s*_*r*_ from ${𝐇}_{s}^{\prime }$
- ${𝐞}_{r}^{+}$: an augmented view of ${𝐞}_{r}$
- ${𝐳}_{𝒯}=\text{Pool}({𝐇}_{𝒯}^{\prime })$: the pooled trajectory embedding (e.g., attention-masked mean pooling)
- ${𝐳}_{i}$, ${𝐳}_{i}^{+}$: anchor and positive trajectory embeddings for contrastive learning

**(2) Trajectory-Trajectory Contrastive Learning (TT):** We apply multiple augmentations to a trajectory ${𝒯}_{i}$ to form anchor-positive pairs $\left({𝐳}_{i},{𝐳}_{i}^{+}\right)$. The augmentations include:

- **Point Masking:** randomly replaces 15% of segments with a [MASK] token
- **Shifting:** rotates trajectory positions to simulate different starting points
- **Truncation:** removes a portion of the end to simulate partial observations

These augmentations introduce diversity and allow the model to focus on core structural patterns. The TT loss is computed using InfoNCE [26]:

$${ℒ}_{\text{TT}}=-\log\frac{\exp(\text{sim}({𝐳}_{i},{𝐳}_{i}^{+})/\tau )}{\exp(\text{sim}({𝐳}_{i},{𝐳}_{i}^{+})/\tau )+\sum_{j\in {𝒩}_{i}}\exp(\text{sim}({𝐳}_{i},{𝐳}_{j}^{-})/\tau )}$$ (9)

**(3) Road-Road Contrastive Learning (RR):** We generate positive pairs $\left({𝐞}_{r},{𝐞}_{r}^{+}\right)$ using:

- **Node Dropping:** randomly zeroes a subset of road embeddings

These perturbations simulate occlusion and promote robustness to missing or noisy map data:

$${ℒ}_{\text{RR}}=-\log\frac{\exp(\text{sim}({𝐞}_{r},{𝐞}_{r}^{+})/\tau )}{\exp(\text{sim}({𝐞}_{r},{𝐞}_{r}^{+})/\tau )+\sum_{k\in {𝒩}_{r}}\exp(\text{sim}({𝐞}_{r},{𝐞}_{k}^{-})/\tau )}$$ (10)

**(4) Trajectory-Road Contrastive Learning (TR):** We align pooled trajectory embeddings ${𝐳}_{𝒯}$ with semantically related road embeddings ${𝐞}_{r}^{\text{mix}}$ generated by:

- **Mixed View Fusion:** interpolating road and trajectory embeddings

The loss promotes alignment between moving behavior and spatial context:

$${ℒ}_{\text{TR}}=-\log\frac{\exp(\text{sim}({𝐳}_{𝒯},{𝐞}_{r}^{\text{mix}})/\tau )}{\exp(\text{sim}({𝐳}_{𝒯},{𝐞}_{r}^{\text{mix}})/\tau )+\sum_{n\in {𝒩}_{𝒯}}\exp(\text{sim}({𝐳}_{𝒯},{𝐞}_{n}^{-})/\tau )}$$ (11)

Masked modeling compels the encoder to reconstruct missing or perturbed input components, encouraging fine-grained understanding of local structure and semantics. Contrastive learning, in contrast, emphasizes global discriminability by separating similar and dissimilar samples in representation space. When combined, these objectives complement each other: masked modeling guides the model to capture detailed spatial-temporal context, while contrastive learning ensures those details contribute to a representation space that preserves semantic relationships. This synergy is especially beneficial in distinguishing hard negatives—cases that are structurally or geographically close but semantically different—by forcing the model to consider both fine details (via reconstruction) and broader context (via contrast).

### Adaptive negative sampling with dual FAISS indexing

To enhance contrastive learning, we introduce an adaptive negative sampling strategy based on FAISS Approximate Nearest Neighbor (ANN) retrieval. Instead of relying solely on minibatch negatives, we maintain two separate FAISS indices—one for trajectory embeddings and one for road segment embeddings. These indices enable efficient retrieval of semantically hard negatives specific to each contrastive task.

The indices are rebuilt periodically during training and used to sample negatives close to the embedding space, but do not overlap semantically with the anchor. This improves the quality of supervision and prevents collapse into trivial solutions. In early epochs, before sufficient representations are learned, the model falls back to standard batch-based negatives.

The dual FAISS-based adaptive sampling mechanism is designed to enhance representation learning by selecting semantically harder negatives from separate trajectory and road embedding spaces. Unlike random negatives, these adaptively selected samples are closer to the anchor in the embedding space, forcing the model to learn finer-grained distinctions and capture subtle spatial-temporal differences. This improves the clustering quality and separation in the representation space.

#### Index construction and update.

Let ${𝐙}_{\text{traj}}=\{{𝐳}_{i}\}$ and ${𝐄}_{\text{road}}=\{{𝐞}_{j}\}$ be the current trajectory and road segment embeddings. Every *E* epoch, we rebuild the indexes:

$$\text{TrajectoryFAISSIndex}\leftarrow \text{FAISS}({𝐙}_{\text{traj}}),\;\text{RoadFAISSIndex}\leftarrow \text{FAISS}({𝐄}_{\text{road}})$$ (12)

These are used to retrieve semantically hard negatives efficiently using L2 similarity.

#### Cold start and warm-up phase.

Before the first index is constructed (epoch <*t*_0_), contrastive losses fall back to minibatch-based negatives:

$${ℒ}_{\text{TT}}^{\text{warmup}}=-\log\frac{\exp(\text{sim}({𝐳}_{i},{𝐳}_{i}^{+})/\tau )}{\sum_{j=1}^{B}\exp(\text{sim}({𝐳}_{i},{𝐳}_{j}^{-})/\tau )}$$ (13)

This bootstraps the representations for meaningful initial ANN sampling.

#### ANN-based sampling per objective.

Once the indices are active, negative samples for each task are queried as follows:

- **TT (Trajectory-Trajectory):** The anchor trajectory ${𝐳}_{i}$ queries the *Trajectory FAISS Index* to get hard negative ${𝐳}_{j}^{-}\notin$ augmentations of ${𝐳}_{i}$.
- **RR (Road-Road):** Road embedding ${𝐞}_{r}$ queries the *Road FAISS Index* to find road nodes that are not neighbors or augmentations.
- **TR (Trajectory-Road):** Trajectory embedding ${𝐳}_{𝒯}$ queries the *Road FAISS Index* to retrieve road segments not included in its path.

To avoid overfitting on nearest negatives, we apply MOCHI-style mixing:

$${𝐞}_{\text{mix}}=\alpha \cdot {𝐞}_{n}^{-}+(1-\alpha )\cdot {𝐞}_{p}^{+},\;\alpha \sim 𝒰(0.5,1.0)$$ (14)

Then the loss over each objective becomes

$${ℒ}_{\text{CL}}^{\text{adaptive}}=-\log\frac{\exp(\text{sim}({𝐳}_{i},{𝐳}_{i}^{+})/\tau )}{\exp(\text{sim}({𝐳}_{i},{𝐳}_{i}^{+})/\tau )+\sum_{n\in {𝒩}_{i}}\exp(\text{sim}({𝐳}_{i},{𝐞}_{n,\text{mix}}^{-})/\tau )}$$ (15)

#### Final objective function.

The full training objective combines all the components:

$${ℒ}_{\text{total}}=\lambda_{1}{ℒ}_{\text{MTM}}+\lambda_{2}{ℒ}_{\text{TT}}+\lambda_{3}{ℒ}_{\text{RR}}+\lambda_{4}{ℒ}_{\text{TR}}$$ (16)

## Experiments

### Dataset

The data sets used in this study are provided by the GAIA project in collaboration with Didi and consist of two months of data on car rides from the cities of Xi’an and Chengdu, China. A summary of these datasets can be found in Table 2. Each dataset includes GPS records for individual trips. Road network data for both cities was gathered from OpenStreetMap, and a map-matching algorithm was employed to align the GPS coordinates to specific road segments. Through this process, trajectories were converted into sequences of road segments. To ensure quality, we filtered out trajectories that included fewer than three road segments or had a duration shorter than one minute. We truncated or padded the trajectories to fixed lengths to ensure uniform input size for model training. Additionally, spatial grids were constructed by aggregating geo-referenced road coordinates into fixed-size tensors, which serve as input to the CNN-based spatial feature extractor in our model.

**Table 2:** Summary of datasets.

Metric	Xian	Chengdu
No. of Road Segments	6,161	6,632
No. of Edges	15,779	17,038
Avg. Trajectory Length (m)	5,880	5,732
Avg. Road Segments per Trip	31.11	30.87

### Evaluation metrics

The evaluation metrics used in all our experiments are summarized as follows:

#### Micro-F1 (Mi-F1) and Macro-F1 (Ma-F1).

Micro-F1 (Mi-F1) and Macro-F1 (Ma-F1) are widely used metrics in classification tasks. Macro-F1 calculates the F1 score for each class individually and then averages these scores, giving equal weight to each class. On the other hand, Micro-F1 takes into account the collective contributions of all classes to compute an overall average F1 score.

#### Root Mean Squared Error (RMSE) and Mean Absolute Error (MAE).

Root Mean Squared Error (RMSE) and Mean Absolute Error (MAE) are metrics commonly used for evaluating regression models. They are calculated as follows:

$$\text{MAE}=\frac{\sum |y_{i}-{\hat{y}}_{i}|}{n}$$ (17)

$$\text{RMSE}=\sqrt{\frac{\sum (y_{i}-{\hat{y}}_{i}{)}^{2}}{n}}$$ (18)

where *y*_*i*_ represents the true value, ${\hat{y}}_{i}$ represents the predicted value for the *i*th sample, and *n* is the total number of samples.

#### Mean Rank (MR) and Hit Ratio@K (HR@K).

Mean Rank (MR) and Hit Ratio@K (HR@K) are widely used metrics for evaluating ranking and recommendation systems. MR calculates the average of all individual ranks and can range from 1 to infinity. HR@K measures the proportion of correct answers that appear within the top *K* entries of the ranking list, with values ranging from 0 to 1. In this experiment, *K* is set to 10.

### Downstream tasks and benchmarks

We conduct four downstream traffic tasks, with two road segment-based tasks and the other two being trajectory-based tasks. We compare our method to several state-of-the-art road and trajectory representation learning methods, as well as graph representation learning methods. Methods designed solely for specific tasks are excluded from the comparison, as we aim to learn robust representations for various tasks. Task-specific methods often include tailored representations and components, resulting in an inconsistent and unfair comparison.

#### Road segment-based tasks.

To assess the representation of road networks, we focus on two main tasks: (1) road label classification and (2) traffic speed prediction.

**Road label classification:** This task is analogous to node classification in graphs. Road-type labels, such as motorways and living streets, are collected from OpenStreetMap. The five most common label types are selected as prediction targets. A classifier composed of a fully connected layer followed by a softmax layer is applied to the road segment representations. The performance is evaluated using Micro-F1 (Mi-F1) and Macro-F1 (Ma-F1) scores.

**Traffic speed prediction:** This is a regression task where the objective is to predict the average speed on each road segment, calculated from trajectory data. A linear regression model is trained using the road representations, and the evaluation is conducted using Mean Absolute Error (MAE) and Root Mean Squared Error (RMSE).

#### Benchmarks for trajectory representation.

We compare our approach to the following road and graph representation methods: Node2vec [6], RFN [?], HRNR [17], JCLRNT [2] and TCRTRL [9].

#### Trajectory-based tasks.

To evaluate trajectory representations, we focus on two main tasks: (1) trajectory similarity search and (2) travel time prediction.

**Trajectory similarity search:** The objective is to identify the most similar trajectory to a given query trajectory from a database. Trajectory representations are used to calculate similarity scores and rank the results in descending order. Performance metrics include Hit Ratio@10 (HR@10) and Mean Rank (MR).

**Travel time prediction:** This task involves predicting the travel time for a given trajectory. The performance is evaluated using Mean Absolute Error (MAE) and Root Mean Squared Error (RMSE).

#### Benchmarks for trajectory representation.

The following methods are used as benchmarks for trajectory representation: Para2vec [27], T2Vec [7], JCLRNT [2] and TCRTRL [9].

### Experimental settings

The training dataset comprises 500,000 trajectories, and we train the model with a batch size of 64 over 20 epochs with a learning rate of 0.001. The model’s architecture is configured with a unified embedding dimension of 128, a GAT hidden dimension of 64 with 4 attention heads, and 2 layers each for the spatial and trajectory transformer encoders. The loss function is weighted with $\lambda_{1}$ = 0.3, $\lambda_{2}$ = 0.1, $\lambda_{3}$ = 0.1, and $\lambda_{4}$ = 0.5. Additionally, the ANN index is updated every 3 epochs. Representation vectors for road segments and trajectories are extracted, all standardized to a dimension of 128, and used in various downstream tasks. The trajectory data set is split into training and evaluation sets based on the date, ensuring that there is no overlap. Training was performed on a machine with a single NVIDIA A4000 GPU (16 GB) and 128 GB RAM. Training took approximately 12 hours per model variant.

### Results and analysis

The simulation results for the four tasks are presented in Tables 3 and 4, with the best results highlighted in bold. Higher values of Mi-F1, Ma-F1, and HR@10 indicate better performance (↑), while lower values of MAE, RMSE, and MR indicate better performance (↓). Our model outperforms other baselines in all tasks and metrics. It effectively captures both the structural topology of road networks through incorporating graph and grid representations, which most other models misses. Masked trajectory modeling helps inject the dynamic movement semantics of trajectories into representations, whereas contrastive learning complements it by learning a more discriminative embedding space. The adaptive negative sampling module further helps to enhance the quality of representations.

**Table 3:** Performance comparison for road label classification and traffic speed inference.

Task	Road Label Classification				Traffic Speed Inference			
	Chengdu		Xian		Chengdu		Xian	
	Mi-F1 ↑	Ma-F1 ↑	Mi-F1 ↑	Ma-F1 ↑	MAE ↓	RMSE ↓	MAE ↓	RMSE ↓
Node2Vec	0.527	0.498	0.583	0.557	7.123	8.998	6.407	8.219
RFN	0.514	0.486	0.574	0.566	6.888	8.772	6.568	8.428
HRNR	0.539	0.526	0.629	0.612	7.031	8.816	6.522	8.447
JCLRNT	0.635	0.628	0.727	0.702	4.687	6.848	5.021	7.083
TCRTRL	0.648	0.637	0.740	0.710	4.575	6.777	4.958	7.007
Proposed MRRT	**0.752**	**0.737**	**0.783**	**0.769**	**2.304**	**3.183**	**2.412**	**3.284**

**Table 4:** Performance comparison for similar trajectory search and travel time estimation.

Task	Similar Trajectory Search				Travel Time Estimation			
	Chengdu		Xian		Chengdu		Xian	
	MR ↓	HR@10 ↑	MR ↓	HR@10 ↑	MAE ↓	RMSE ↓	MAE ↓	RMSE ↓
Para2vec	218	0.257	280	0.209	222.7	307.4	247.3	351.8
T2Vec	46.5	0.789	38.3	0.809	167.5	245.5	208.2	314.7
JCLRNT	8.90	0.922	9.59	0.909	124.1	184.8	166.3	244.7
TCRTRL	8.67	0.926	9.18	0.911	122.2	180.5	164.2	243.8
Proposed MRRT	**7.70**	**0.935**	**8.64**	**0.918**	**120.2**	**178.5**	**162.2**	**241.8**

In comparison to baseline models, we also evaluated the computational costs of MRRT relative to node2vec and JCLRNT on an NVIDIA A4000 GPU. Node2vec, as a lightweight baseline, has a small model size of approximately 780,800 parameters and achieves inference times under 1 ms for a single trajectory, but it lacks the expressiveness needed for complex spatio-temporal trajectory modeling. JCLRNT serves as a stronger baseline with its hybrid GAT and Transformer architecture, containing about 1.08 million parameters and enabling efficient inference within 2–5 ms. Our proposed MRRT framework employs a larger model (around 1.45 million parameters), integrating CNN-based spatial fusion and cross-attention mechanisms to learn richer node representations from raw features. This results in improved accuracy on challenging tasks at a moderate increase in inference time of 5–10 ms. Importantly, MRRT scales effectively with larger networks and longer trajectories by leveraging batched inference and ANN-based adaptive negative sampling, providing a practical balance between computational cost and model performance.

### Ablation study

To evaluate the contribution of each proposed component to our architecture, we conducted an ablation study (shown in Fig 3) on four downstream tasks: road classification, traffic speed inference, travel time estimation, and search for trajectory similarity. We consider four variants of the model in increasing order of complexity.

**Fig 2:**
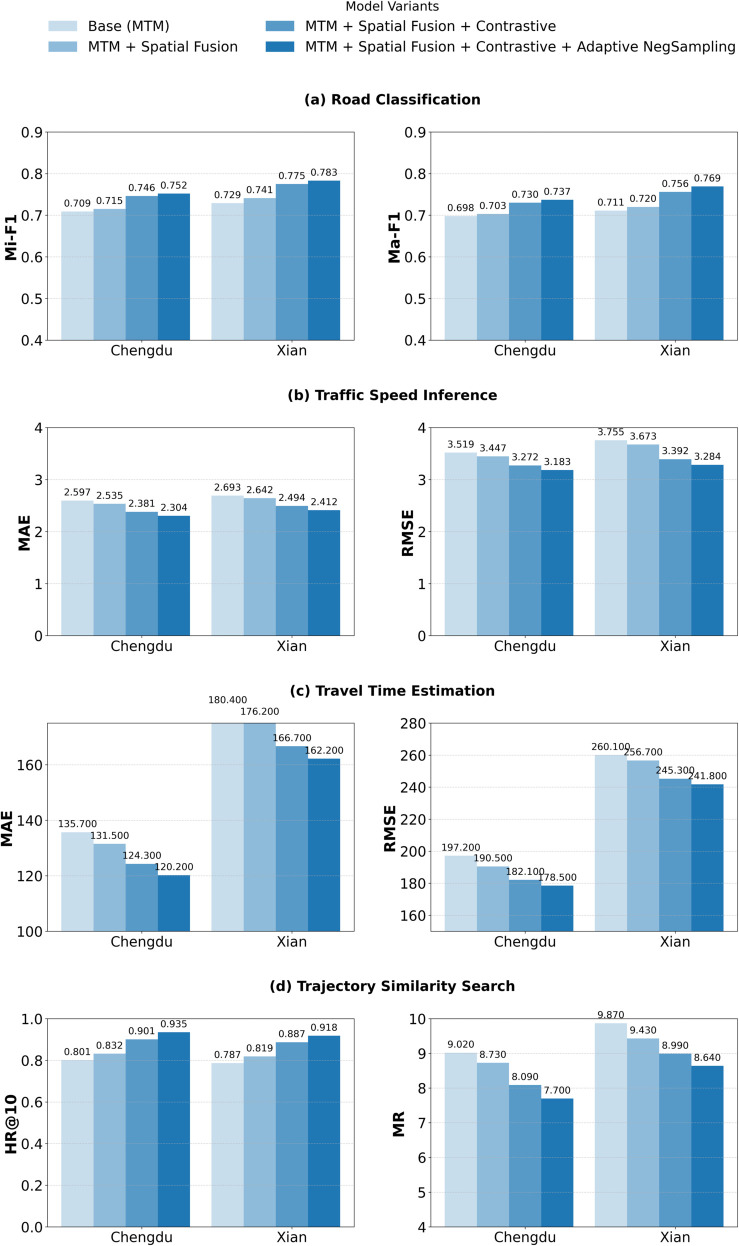
Ablation study results across the downstream tasks.

**Fig 3:**
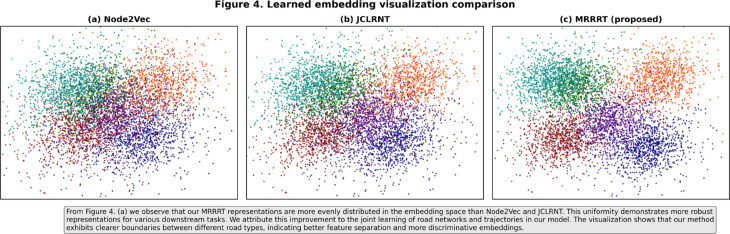
Embeddings of the road segment representations learned by (a) Node2Vec, (b) JCLRNT and (c) the proposed MRRT method.

- **Base (MTM)**: The core sequence modeling architecture trained with the masked trajectory modeling (MTM) objective only.
- **+ Spatial Fusion**: Introduces trajectory-aware spatial encoding to capture local and global road structure.
- **+ Contrastive Learning**: Adds a contrastive objective to learn more discriminative representations by enforcing consistency between similar trajectories.
- **+ Adaptive Negative Sampling**: Enhances contrastive loss by dynamically selecting harder negative examples during training.

Fig 2 presents ablation results in both the Chengdu and Xian datasets for each task. In general, we observe that all components provide consistent improvements in all evaluation metrics. Spatial fusion helps to achieve early gains by integrating the topological context. Contrastive learning significantly enhances representational quality, and adaptive sampling further sharpens decision boundaries. The whole model achieves the best performance across all tasks and metrics, demonstrating the complementary nature of each component.

### Parameter sensitivity analysis

We fixed $\lambda_{2}$ and $\lambda_{3}$ to 0.1 based on their relatively smaller but consistent contribution to performance. This leaves $\lambda_{1}\;+\;\lambda_{4}=0.8$, which we varied to assess the trade-off between the masked trajectory modeling (MTM) and road-trajectory contrastive learning. We found that allocating a larger portion ($\lambda_{4}=0.5$) to the road-trajectory contrastive loss leads to stronger alignment between trajectory and road embeddings, which benefits both similarity search and road classification tasks. In contrast, setting $\lambda_{1}$ too high (e.g., $\lambda_{1}=0.5$, $\lambda_{4}=0.3$) resulted in degraded performance, suggesting that excessive emphasis on MTM encourages overfitting to trajectory continuity while under-utilizing structural alignment. The chosen setting ($\lambda_{1}=0.3$, $\lambda_{4}=0.5$) offered the best trade-off across all tasks by preserving trajectory semantics while maximizing cross-view consistency. About mask ratio, higher masking increases the challenge and may encourage the model to learn more robust representations, but too high a ratio can obscure essential context, degrading performance. In our experiments, a moderate ratio (around 30–40%) yielded the best results. Regarding index update frequency for FAISS-based adaptive sampling, frequent updates improve negative sampling quality but add overhead. Empirically, updating every few epochs (e.g., 3-5) provides a good trade-off between computational cost and retrieval accuracy.

### Visualization

To visually assess the quality and separability of the learned embeddings, we project the road segment representations from Node2Vec, JCLRNT, and our proposed MRRT model using t-SNE [28]. As shown in Fig 3, the visualization reveals significant differences in the learned representations. The embeddings from the traditional Node2Vec approach appear heavily clustered with poor separation between different road types, suggesting a less discriminative representation. In contrast, both JCLRNT and our MRRT model produce embeddings with clearer, more distinct cluster boundaries, indicating that these joint learning frameworks are more effective at capturing the semantic differences between road segments. Notably, the embeddings from our MRRT model are more uniformly distributed and exhibit slightly more compact and better-separated clusters compared to JCLRNT. This improved feature separation demonstrates that our multi-objective, self-supervised framework learns more robust and discriminative representations, which contributes to its superior performance on a variety of downstream tasks.

## Applications of MRRT

MRRT produces unified embeddings for road segments and trajectories that capture structural, spatial, and temporal semantics, and we have already validated their utility in four diverse downstream tasks: road classification, traffic speed inference, travel time estimation, and trajectory similarity search. These tasks reflect real-world needs: for example, trajectory similarity can power abnormal driving detection or route recommendation; travel-time estimation is crucial for logistics and trip planning; and road classification aids in infrastructure monitoring. Technically, the learned embeddings can be directly fed into lightweight task-specific layers, such as a regression head for speed prediction or a contrastive similarity module for retrieval, demonstrating plug-and-play flexibility. In practice, routing algorithms can embed the MRRT road features in learned cost functions, allowing a shallow neural layer to estimate edge weights that represent travel efficiency or congestion likelihood. Clustering the embeddings can highlight traffic hotspots or high-incident zones, which supports adaptive signal control and congestion mitigation. MRRT’s trajectory embeddings can also feed into reinforcement learning agents (e.g., for eco-routing or signal scheduling), replacing handcrafted state features with compact, generalizable representations. Furthermore, embeddings can be plugged into simulation environments like SUMO or MATSim to enhance behavioral modeling. These examples illustrate how MRRT’s architecture is not only effective for benchmark tasks but also readily transferable to operational decision-making in transportation systems. In addition to these, MRRT can serve as a plug-and-play representation layer for a wide range of domain-specific engineering objectives. Since MRRT learns semantically rich embeddings of road segments and trajectories, these can be easily integrated into specialized downstream models by attaching lightweight task-specific heads. For instance, in traffic prediction, road-level embeddings from MRRT can be combined with historical incident labels and fed into a classification layer for supervised training. This could help reduce time in traffic prediction. Similarly, in maintenance prioritization, the embeddings of the trajectories could be clustered to identify the underused or overburdened segments, guiding the targeted maintenance strategies.

## Discussion

### On cross-city generalization

Our current evaluation focused on assessing the model’s generalization across diverse tasks within the same city, which was the primary goal of this study. We believe that our model, as a pre-training framework, is theoretically well-suited to provide a general representation for road networks and trajectories, which contributes to its strong performance across various downstream tasks. While we acknowledge that applying the model to a new city requires updating the road and trajectory graphs and potentially fine-tuning some higher-level layers, this is a standard challenge in transfer learning. We believe the cost of this adaptation would be relatively low, and we consider cross-city transfer to be a promising and important future direction for validating our model’s universal structural modeling and generalization ability.

### Robustness to data imperfections and imbalance

In real-world deployment, GPS trajectories often suffer from low sampling rates, signal drift, or positioning errors, leading to imperfections in map-matched sequences. Furthermore, datasets can be highly imbalanced, with some movement patterns occurring far more frequently than others. MRRT addresses these challenges through several design properties. First, a transformer-based sequence model with learnable temporal embeddings enables the model to infer contextual signals even when segments are noisy or incomplete. This is reinforced during training through augmentations such as segment masking, truncation, and shifting, which simulate common real-world distortions and implicitly regularize the model to tolerate partial and imprecise inputs. Second, our contrastive learning losses and hard negative sampling strategies encourage the model to learn invariant features that capture underlying movement behavior and road semantics, rather than overfitting to exact, potentially noisy sequences. This approach is particularly effective for handling data imbalance by dynamically selecting diverse training pairs. Although we did not perform a dedicated sensitivity analysis to extreme noise or imbalance, we acknowledge that these are critical considerations. Future work could explore more advanced noise-aware training strategies, integrate confidence scores from the map-matching stage, or adopt curriculum learning to handle such cases more effectively.

### Impact of training data size

Our experiments demonstrate that training MRRT with more trajectories consistently improves performance, especially in the early stages. The model initially benefits significantly from increasing data, indicating that it effectively learns from diverse patterns and reduces bias. However, the performance gain gradually degrades beyond a certain point, reflecting a saturation in representational improvement. In our setting, approximately 500,000 trajectories provided an optimal trade-off between accuracy and training cost. This suggests that MRRT can still be trained effectively in mid-sized or data-scarce cities, though some performance degradation may occur with fewer trajectories.

## Conclusion

This paper proposes a unified framework for jointly learning representations of road networks and trajectories through self-supervised multi-objective learning. By integrating graph-based and grid-based representations for road network representations along with employing temporal encodings in sequential trajectory representations, our MRRT model effectively captures both the structural topology of road networks and the dynamic movement semantics of trajectories. A key contribution of our method lies in the use of contrastive learning objectives across multiple views: trajectory-trajectory, road-road, and trajectory-road relations, supported by a novel adaptive negative sampling strategy. This design ensures that the model is progressively challenged with semantically hard negatives throughout training, significantly improving the quality of the learned representations. Future directions include incorporating more modalities of data (images and textual descriptions), modeling cross-city transferability, etc., and testing the performance of MRRT on highly imbalanced or heavily corrupted data. Although we rely on reasonably accurate map-matched data, we acknowledge that extreme noise or imbalance could impact performance. In such cases, strategies like gradually training on cleaner to noisier data (curriculum learning) can be explored in future work.
